# A Viral microRNA Down-Regulates Multiple Cell Cycle Genes through mRNA 5′UTRs

**DOI:** 10.1371/journal.ppat.1000967

**Published:** 2010-06-24

**Authors:** Finn Grey, Rebecca Tirabassi, Heather Meyers, Guanming Wu, Shannon McWeeney, Lauren Hook, Jay A. Nelson

**Affiliations:** 1 Vaccine and Gene Therapy Institute, Oregon Health & Science University, Portland, Oregon, United States of America; 2 Knight Cancer Institute, Oregon Health & Science University, Portland, Oregon, United States of America; 3 OCTRI, Oregon Health & Science University, Portland, Oregon, United States of America; University of Alabama at Birmingham, United States of America

## Abstract

Global gene expression data combined with bioinformatic analysis provides strong evidence that mammalian miRNAs mediate repression of gene expression primarily through binding sites within the 3′ untranslated region (UTR). Using RNA induced silencing complex immunoprecipitation (RISC-IP) techniques we have identified multiple cellular targets for a human cytomegalovirus (HCMV) miRNA, miR-US25-1. Strikingly, this miRNA binds target sites primarily within 5′UTRs, mediating significant reduction in gene expression. Intriguingly, many of the genes targeted by miR-US25-1 are associated with cell cycle control, including cyclin E2, *BRCC3*, *EID1*, *MAPRE2*, and *CD147*, suggesting that miR-US25-1 is targeting genes within a related pathway. Deletion of miR-US25-1 from HCMV results in over expression of cyclin E2 in the context of viral infection. Our studies demonstrate that a viral miRNA mediates translational repression of multiple cellular genes by targeting mRNA 5′UTRs.

## Author Summary

Regulation of gene expression is as important as the genes themselves in determining the diverse array of living creatures we see in nature. Recently, scientists have discovered a whole new level of gene regulation through the actions of small molecules called microRNAs (miRNAs). It is currently thought that miRNAs regulate gene expression primarily through binding to target sites within the 3′UTR of mRNAs. Here we identify a population of cellular genes that are targeted by a virally encoded miRNA. Many of the genes are related to cell cycle control, suggesting that the viral miRNA is targeting genes within a related pathway. In contrast to most miRNAs, this miRNA inhibits gene expression through binding to target sites within the 5′UTRs, suggesting that viral miRNAs may target genes through mechanisms divergent from cellular miRNAs.

## Introduction

The recent discovery of a new class of regulatory genes known as microRNAs (miRNAs) has resulted in a paradigm shift in gene regulation research. miRNAs are small single-stranded RNA species of approximately 20–24 bases in length that regulate gene expression through post transcriptional mechanisms [1]. Expression of miRNAs is thought to be ubiquitous among multicellular eukaryotes [2]. In addition to eukaryotic miRNAs, more than 100 viral miRNAs have been identified, almost all of which are expressed by herpesviruses [3]. Targets for the majority of viral miRNAs are currently unknown due to the difficulty involved in identifying novel target transcripts. This remains one of the major challenges in elucidating the function of miRNAs. However, recent reports have begun to elucidate the various roles of viral miRNAs. These include blocking apoptosis, immune evasion and regulation of viral replication through targeting of both cellular and viral gene expression [4], [5].

HCMV, a member of the beta-herpesvirus sub family, encodes at least 11 miRNAs [6]–[8]. Previously, we demonstrated that the HCMV encoded miRNA, miR-UL112-1, targets a number of the virus's own genes, including the immediate early transactivator IE72 which is essential for driving acute replication of HCMV [9]. In addition, miR-UL112-1 targets the cellular gene MICB, resulting in protection against recognition by natural killer cells [10]. miR-UL112-1 may therefore play an important role in establishing and maintaining viral latency and persistence through regulation of viral gene expression and subversion of host antiviral pathways. Indeed, consensus is beginning to emerge that herpesvirus miRNAs may, in general, be important in establishing and maintaining viral persistence [9], [11]–[13].

Current studies indicate that miRNA targeting in mammalian cells occurs predominantly through binding to sequences within 3′UTRs [14]–[16]. The reason for this bias is unclear, although a recent study demonstrated that miRNA-mediated repression of a reporter construct was less efficient when the target site was placed in the ORF compared to the 3′UTR [17]. In contrast, inhibition of gene expression through targeting the 5′UTR has been demonstrated, at least in the context of an artificial reporter construct, indicating that miRNA targeting of 5′UTRs is possible [18]. However, statistical analysis of conserved miRNA target sequences and global biochemical screens have demonstrated that mammalian miRNA target sites rarely occur within 5′UTRs [14]–[16], [19]. The role of 5′UTRs in miRNA regulation is further complicated by a study that found miR-10a induces, rather than inhibits, protein expression through binding to 5′UTRs of cellular transcripts [20]. In addition, binding of the liver specific miRNA, miR-122 to the 5′UTR of Hepatitis C (HCV) genome is required for virus replication [21]. These studies suggest a model in which binding to the 5′UTR results in mechanistic effects divergent from 3′UTR binding.

Here, we identify cellular transcript targets of one of the most highly expressed HCMV encoded miRNAs, miR-US25-1, using a recently developed biochemical approach called RISC immunoprecipitation. Strikingly, the majority of identified transcripts contained miR-US25-1 target sites within the 5′UTR rather than the 3′UTR. The target transcripts include a number of genes associated with cell cycle control, including cyclin E2, as well as histone proteins, suggesting that miR-US25-1 is targeting functionally related genes. Crucially, we demonstrate that targeting of cyclin E2 by miR-US25-1 occurs in the context of HCMV infection and results in inhibition of cyclin E2 protein expression.

## Results

### Identification of cellular targets of miR-US25-1 through RISC immunoprecipitation

**Figure 1:**
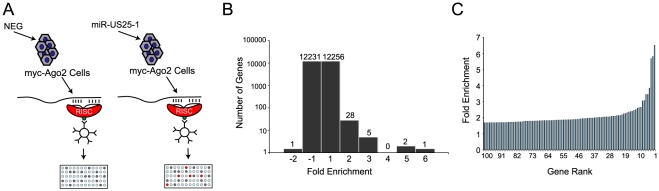
RISC-IP analysis of HCMV encoded miRNA, miR-US25-1. (a) Schematic representation of c-myc tagged Ago2 pull downs. (b) Distribution of fold enrichment and (c) enrichment levels of top 100 transcripts following RISC-IP analysis using c-myc tagged Ago2 approach.

Identifying target transcripts is still one of the main challenges in determining the functional role of miRNAs. Although bioinformatic strategies have proven useful, they are limited by high false positive rates. Due to a lack of effective approaches, target transcripts for the majority of viral miRNAs, and miRNAs in general remain unknown. Alternative experimental approaches for the identification of miRNA targets that do not completely rely on bioinformatic predictions are therefore desirable. Recently, approaches for miRNA target identification have been devised that rely on immunoprecipitation of the RISC complex and the associated target transcripts [22], [23]. As part of the RISC complex, miRNAs bind to target transcripts and form stable interactions. Using a HEK293 cell line that expresses a tagged component of RISC (Argonaute 2 (Ago2) tagged with myc epitope) these complexes can be immunoprecipitated and targeted transcripts identified by microarray analysis (Figure 1a). This approach was used to identify cellular targets of one of the most highly expressed HCMV miRNAs, miR-US25-1. The Ago2 tagged cell line was transiently transfected with a construct encoding the full-length pre-miRNA of miR-US25-1, under the control of the human U6 polymerase III promoter. Three days post transfection cells were harvested and RISC complexes were immunoprecipitated with a myc-epitope specific antibody. mRNA levels within the immunoprecipitated complexes as well in whole cell lysates were quantified by microarray analysis. As previously described [22], [23], association of a specific mRNA with the RISC complex is represented by quantitative enrichment of the mRNA in the immunoprecipitated fraction relative to the total (whole cell) fraction. In order to determine which targets are specifically associated with miR-US25-1, fold enrichment of transcripts immunoprecipitated from miR-US25-1 transfected cells was compared to values from cells transfected with a vector expressing a negative control miRNA. miR-US25-1 specific targets are only expected to be enriched in cells expressing miR-US25-1. Transcripts were then ranked according to the level of enrichment with the highest enriched transcripts considered potential targets of miR-US25-1 (Table S1). Over all, the results indicate that miR-US25-1 RISC complexes associated with a relatively small population of transcripts (Figure 1b and c) and fold changes were skewed towards enrichment as would be expected if miR-US25-1 were targeting a specific population of genes. The majority of transcripts were not enriched, showing enrichment levels close to 1 (Figure 1b). Thirty-six transcripts showed greater than 2-fold enrichment, while only 1 transcript was reduced by more than 2-fold. The highest level of enrichment was 6.5 fold.

**Figure 2:**
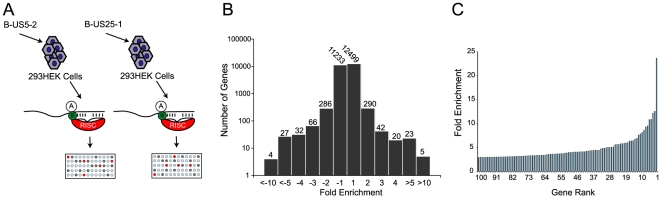
RISC-IP analysis of HCMV encoded miRNA, miR-US25-1. (a) Schematic representation of biotin pull downs. (b) Distribution of fold enrichment and (c) enrichment levels of top 100 transcripts following RISC-IP analysis using biotin approach.

To increase confidence in target identification, a second, parallel approach was used in which a biotinylated synthetic mimic of miR-US25-1 was transfected into HEK293 cells and miR-US25-1 specific miRNA protein complexes (miRNPs) were isolated using streptavidin bead pull downs (Figure 2a and Table S2). In contrast to the previous approach this should only pull down direct targets of miR-US25-1. Again, most genes showed little or no enrichment with a relatively small population of transcripts showing exponential increase in enrichment (Figure 2b and c). However, the levels of enrichment were much higher following biotin isolation, reaching a maximum of 23.6 fold. In this case fold changes did not skew towards enrichment as miR-US25-1 was compared to a second HCMV miRNA, miR-US5-2, rather than a negative control. Transcripts showing a negative enrichment ratio, likely represent targets of miR-US5-2.

The two data sets were compared to determine whether the same genes were identified by both RISC pull down approaches. As the enrichment levels in the biotin approach were higher than those found with the myc-Ago2 approach, averaging the enrichment levels would result in bias towards the biotin data set. To avoid this bias, the data sets were combined using rank sum analysis. Transcripts were assigned a rank based on the comparative level of enrichment (highest enriched  =  rank 1, lowest  =  rank 24526) then the average rank between the myc-Ago2 approach and the biotin approach was calculated for each gene. Although differences existed in the rankings of the two data sets (for example *TRIM28* was ranked 1^st^ in the c-myc approach, but 188^th^ in the biotin approach) a population of transcripts were enriched by both approaches. Fifteen of the top 20 genes showed greater than 2 fold enrichment by both approaches, giving high confidence that these transcripts were likely targets of miR-US25-1. Table 1 shows the top 20 ranked genes by rank sum analysis including a summary description of their function and the enrichment levels by each approach. A number of these targets are involved in cell cycle control (cyclin E2, *BRCC3*, *PSMA4* and *EID1* [24]–[27]) and tumor progression (*ASRGL1*, *CD147*, *MAPRE2* and *ASPSCR1* [28]–[31]), while three of the targets encode histone genes (*LOC440093*, *H3F3B*, *HIST2H4A*), indicating that miR-US25-1 targets functionally related genes.

**Table 1:** Top 20 ranked cellular targets of miR-US25-1.

	myc-Ago2		Biotin					
Target ID	Enrichment	Rank	Enrichment	Rank	Rank ave	Target Site	DEFINITION	
CCNE2	5.8	2	12.2	3	2.5	+^1^	G1/S cyclin E2	
BCKDHA	3.1	8	23.7	1	4.5	+^2^	branched chain keto acid dehydrogenase	
LOC440093	5.7	3	6.9	12	7.5	+^1^	similar to H3 histone, family 3B	
POP4	2.4	14	6.4	15	14.5	-	ribonuclease P/MRP subunit	
ATP6V0C	3.8	4	4.4	33	18.5	+^3^	ATPase, H+ transporting, lysosomal	
ASRGL1	2.1	29	7.9	10	19.5	+^1^	asparaginase like 1	
BRCC3	2.0	39	12.6	2	20.5	+^2^	G2/M transition DNA damage checkpoint	
H3F3B	2.5	12	4.4	36	24	+^1^	H3 histone, family 3B	
PSMA4	3.1	7	4.0	50	28.5	+^1^	proteasome subunit	
HIST2H4A	2.1	30	5.0	28	29	+^1^	histone cluster 2, H4a	
CD147	1.8	68	9.5	6	37	+^1^	Tumor progression	
MAPRE2	2.1	26	4.0	48	37	+^1^	Homology to APC	
SGSH	2.1	24	3.6	58	41	+^2^	degradation of heparan sulfate	
CCDC58	1.8	70	6.8	13	41.5	-	coiled-coil domain containing 58	
BLVRA	1.8	77	5.7	22	49.5	+^1^	biliverdin reductase A	
NUCB2	1.9	56	4.1	43	49.5	+^1^	nucleobindin 2	
EID1	3.4	6	3.0	94	50	+^1^	EP300 interacting inhibitor of differentiation	
ASPSCR1	2.1	25	3.1	80	52.5	-	alveolar soft part sarcoma	
SGK	1.7	89	4.4	35	62	-	serum/glucocorticoid regulated kinase	
DBN1	2.3	19	2.9	106	62.5	-	actin binding	

Top 20 transcripts enriched by miR-US25-1 following RISC immunoprecipitation. Enrichment was determined by dividing total RNA levels by the levels of RNA detected following IP. Fold enrichment represents enrichment of miR-US25-1 transcripts compared to negative control and is representative of 2 biological replicates. Superscript numbers attached to gene names indicate miR-US25-1 target sites within these transcripts as follows;

1 = 7 to 8 base target within the 5′UTR,

2 = target site without strict seed sequence complementarity within 5′UTR,

3 = 7 base target within coding region (see Figure S1 for details).

### miR-US25-1 targets sites within the 5′UTR of transcripts

**Figure 3:**
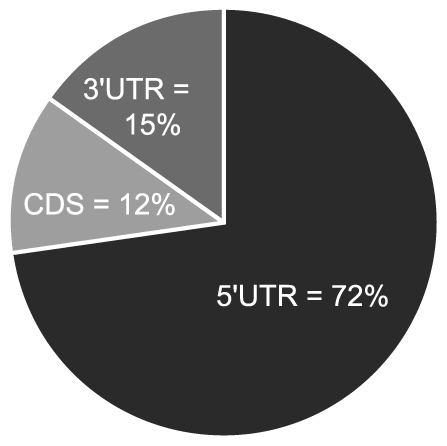
Majority of miR-US25-1 target sites reside within 5′UTRs. Pie chart shows the percentage of miR-US25-1 seed matches within the 5′UTR, ORF or 3′UTR of top 50 enriched transcripts.

If the observed enrichment were due to direct targeting by miR-US25-1, the identified population of transcripts would be expected to contain binding sites for the miRNA. Binding of the 5′ end of the miRNA, specifically nucleotides 1–8, known as the seed sequence, are thought to be particularly important [1], [32], [33]. Therefore, the transcripts in the database were searched for seed sequence matches complimentary to nucleotides, 1–7, 2–8 and 1–8 of miR-US25-1. The number of seed matches within the top 50 enriched transcripts from Table S3 was then compared to the number of matches within the rest of the gene list, thereby determining whether genes highly enriched, were more likely to contain predicted miR-US25-1 target sites. Strikingly, miR-US25-1 seed matches were significantly overrepresented within the 5′UTRs of enriched genes (Figure 3 and Table 2). Twelve of the top 20 genes shown in Table 1 contained at least one 7 base seed match within the 5′UTR. In addition, a further 3 genes contain target sites within the 5′UTRs that do not strictly adhere to Watson-Crick base pairing within the seed region. One gene, *ATP6V0C*, contained a 7 base target within the coding region, 100 bases down stream of the 5′UTR (Table 1 and Figure S1). Crucially the number of target sites within the top 50 genes increased from 19 and 14 with the biotin and c-myc approach individually, to 24 target sites in the combined data set, providing additional evidence that the combined approach provides a more robust method of identifying target transcripts.

**Table 2:** Over representation of seed sequence target sites within top 50 enriched transcripts.

Seed region	Target	5′ Match	5′ P-value	3′ Match	3′ P-value
2–8	GAGCGGU	14	1.2e-14	1	0.36
1–7	AGCGGUU	10	5.5e-11	4	2.5e-3
1–8	GAGCGGUU	5	8.0e-7	1	0.091
2–8 G:U	G*G*GCGGU	3	0.13	1	0.55
2–8 G:U	GAG*U*GGU	1	0.37	3	0.51
2–8 G:U	G*G*G*U*GGU	0	1.0	1	0.98
1–7 G:U	*G*GCGGUU	0	1.0	0	1.0
1–7 G:U	AG*U*GGUU	1	0.33	1	0.98
1–7 G:U	*G*G*U*GGUU	1	0.44	0	1.0

Statistical overrepresentation of miR-US25-1 seed sequences in top 50 enriched genes. Targets represent either nucleotides 1–7, 2–8 or 1–8 of miR-US25-1 seed region. Also shown are the same targets allowing for potential G:U base pairing (italicized).

**Figure 4:**
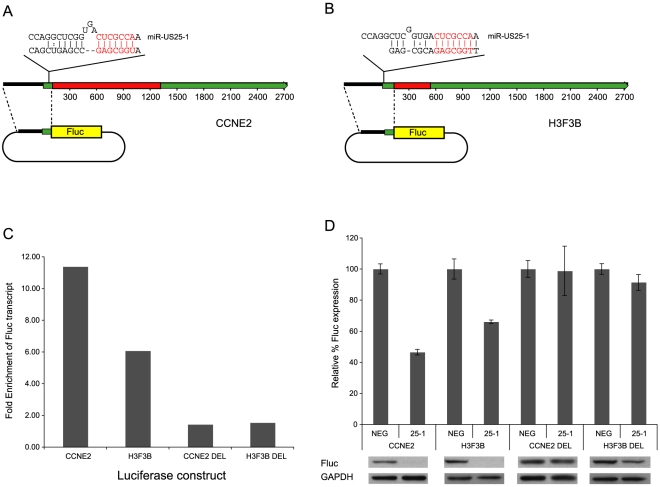
miR-US25-1 represses gene expression. (a) Schematic representation of miR-US25-1 target transcripts, cyclin E2 and (b) H3F3B. Red boxes represent the open reading frames, green boxes the UTRs. The position of the target site within the 5UTR is indicated as well as the predicted binding between miR-US25-1 and target sites. The seed region is highlighted in red. (c) 5′UTR and promoter region of cyclin E2 (CCNE2) and H3F3B were cloned upstream of a luciferase reporter construct. Cells were cotransfected with firefly luciferase (Fluc) constructs and either miR-US25-1 expression plasmid or a negative control plasmid. Following RISC-IP analysis, levels of luciferase transcript were analyzed by RT-PCR and enrichment determined by comparing IP levels with total levels. Target sites were replaced with an EcoRI site to create deletion constructs. Bars represent the fold increase in enrichment following miR-US25-1 expression compared to control. Transcript levels were normalized to GAPDH. (d) Luciferase constructs described above were cotransfected with renillin luciferase plasmid and either miR-US25-1 expression plasmid (US25-1) or the control plasmid (NEG). Luciferase activity was normalized to renillin levels then calculated as percentage of the negative control, which was set to 100%. Error bars indicate s.d. from 3 independent experiments. Equivalent western blots for each transfection is shown below luciferase graph indicating Fluc protein levels.

To confirm that enrichment of identified transcripts was due to binding of miR-US25-1 to the 5′UTR, the 5′UTRs and ∼500 bases of upstream genomic sequence of two of the top target genes, cyclin E2 and *H3F3B*, were cloned in front of a luciferase reporter construct (Figure 4a and b). These constructs were cotransfected into c-myc tagged Ago2 expressing 293 cells with plasmids expressing either miR-US25-1 or the negative control plasmid. RISC-IP analysis was conducted as described above, with levels of luciferase transcript measured using specific RT-PCR primers to the coding region of the reporter gene. Figure 4c shows miR-US25-1 expression resulted in enrichment of luciferase transcript, indicating that the 5′UTRs are indeed sufficient for miR-US25-1 binding. Deletion of the identified seed sequence targets from the 5′UTRs resulted in a loss of enrichment, confirming that the 5′UTR sequences are sufficient and that the target sites are necessary for miR-US25-1 binding.

### Targeting of 5′UTRs by miR-US25-1 results in decreased gene expression

To determine the effect on gene expression of miR-US25-1 binding on the identified 5′UTR targets, luciferase assays were conducted using the 5′UTR constructs described above. In each case expression of miR-US25-1 resulted in a significant reduction in luciferase activity and protein levels (Figure 4d). miR-US25-1 regulation was dependent on the cloned 5′UTRs and the seed target sites as deletion of these target sites rescued expression of luciferase. Expression of miR-US25-1 appears to have resulted in a greater reduction of luciferase protein as determined by western blot, compared to luciferase activity. We speculate that the reduction in luciferase activity is not linearly reflecting the reduction in actually protein levels, possible due to enzymatic nature of the luciferase assay. Direct measurement of luciferase protein may therefore be a more sensitive measure of miRNA regulation.

### miR-US25-1 regulates expression of cyclin E2 and TRIM28 during viral infection

**Figure 5:**
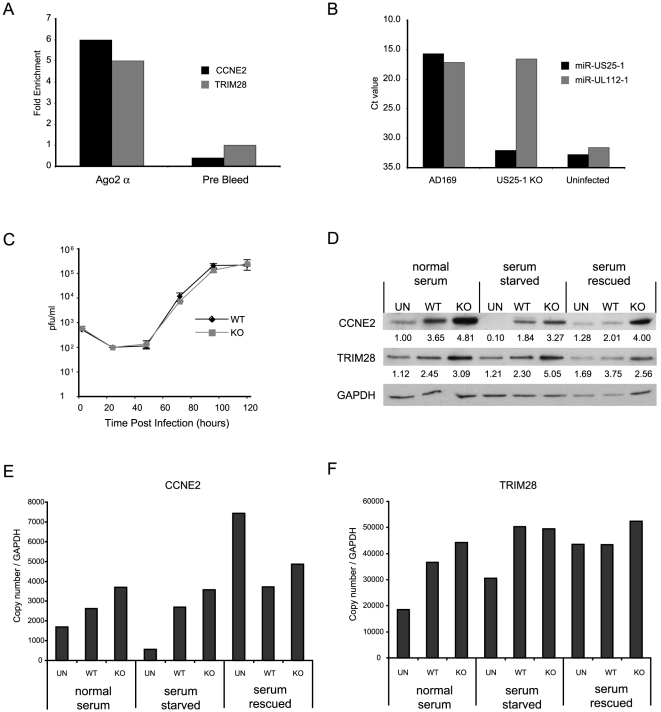
miR-US25-1 targets 5′UTR's in context of viral infection. (a) RISC-IP analysis was conducted on uninfected human primary fibroblast cells or cells infected with HCMV using a direct Ago2 antibody. Results show levels of enrichment of cyclin E2 or TRIM28 transcript from infected cells compared to uninfected cells. RISC-IP was also conducted using pre-bleed antibody derived from rabbits before antigen inoculation. (b) miR-US25-1 was deleted from HCMV. Levels of miR-US25-1 and miR-UL112-1 were determined by RT-PCR analysis following infection of human primary fibroblast cells with either wild type HCMV or the knock out virus. RNA from uninfected cells is used as a negative control. (c) Viral growth of miR-US25-1 knock out virus was compared to wild type HCMV following low (MOI of 0.5) or high (MOI of 10) multiplicity infection of human primary fibroblast cells. Cells plus supernatant were collected at indicated times and assayed on primary human fibroblast cells by limiting dilution (d) Levels of cyclin E2 and TRIM28 protein were determined following high multiplicity infection (MOI of 10) of human primary fibroblast cells with either wild type virus or miR-US25-1 knock out virus. Cells were either grown in normal serum conditions, serum starved conditions or serum starved cells with serum replaced 10 hours prior to harvest. Cells were harvested 72 hours post infection. Relative densities of bands normalized to GAPDH are shown below each lane. Total RNA was also isolated and transcript levels for cyclin E2 (e) and TRIM28 (f) determined by RT-PCR. Transcript copy number was normalized to GAPDH levels.

Although these results confirm that miR-US25-1 can bind to a specific population of cellular transcripts, it is important to determine whether these genes are targeted in the context of a viral infection. HEK293 cells are not permissive to HCMV and have a different gene expression profile than cells that are HCMV permissive. To enable RISC-IP analysis of permissive cell lines, a direct antibody to Ago2 was generated using a peptide from the N-terminus of the protein. This antibody was shown to efficiently recognize endogenous Ago2 (Figure S2a and b). RISC complexes were immunoprecipitated from either uninfected human primary fibroblast cells or cells infected with HCMV. The associated RNA was isolated and subjected to RT-PCR analysis using primers specific to the top target cyclin E2 and TRIM28. Although TRIM28 was not in the top 20 targets shown in Table 2, it was the top target following immunoprecipitation of tagged Ago2 from cells transfected with the pSIREN construct (Table S1). As shown in Figure 5a, cyclin E2 and *TRIM28* were effectively enriched from cells infected with HCMV compared to the uninfected control cells. In addition, immunoprecipitation using a pre-bleed control serum, which is not expected to pull down Ago2, did not result in enrichment, indicating that the effect was specifically due to association with RISC complexes.

To determine the specific effects of miR-US25-1 on the expression of target proteins in the context of viral infection, miR-US25-1 pre-miRNA coding region was deleted from the virus. As shown in Figure 5b, successful disruption of miR-US25-1 expression was confirmed by RT-PCR analysis. Cells infected with wild type HCMV express high levels of both miR-US25-1 and miR-UL112-1, whereas miR-US25-1 levels were below background in cells infected with the knockout virus. miR-UL112-1 levels were equivalent to wild type levels indicating efficient infection with the miR-US25-1 knockout virus. Low (MOI 0.5 – Figure 5c) and high (MOI 10 – supplementary Figure S2c) multiplicity growth curve analysis show the knockout virus was able to replicate with wild type kinetics in human primary fibroblast cells. The effects of virally expressed miR-US25-1 on two top targets, cyclin E2 and *TRIM28*, were determined by western blot analysis. As cyclin E protein levels are regulated throughout the cell cycle, various serum conditions were used to look at the effects of virus infection during normal cycling populations, populations arrested by serum starvation and cells induced from a resting state using replacement of serum. Western blot analysis indicates that serum starvation effectively repressed cyclin E2 expression as expected and serum rescue resulted in resumption of cyclin E2 expression. Furthermore, as is the case with cyclin E1, cyclin E2 levels were induced by HCMV infection in all serum conditions. Figure 5d also shows a clear increase in expression of cyclin E2, and to a lesser extent *TRIM28*, in cells infected with the miR-US25-1 knock-out virus compared to wild type infected cells, demonstrating that miR-US25-1 reduces the expression of these target genes. Time course experiments show that expression of cyclin E2 was equivalent between wild type and KO virus infection 24 hours post infection, and regulation by miR-US25-1 does not occur until approximately 48 hours post infection (supplementary Figure S2d). This concurs with previous studies showing levels of miR-US25-1 increase during the progression of the viral infection and suggests that miR-US25-1 levels at 24 hours post infection are not high enough to produce measurable effects on cyclin E2 protein levels [7]. A slight increase (approximately 1.5 fold) was observed in RNA levels of cyclin E2 and *TRIM28*, consistent with previous reports that miRNA targeting can cause moderate decreases in transcript levels (Figure 5e and f). Following serum rescue, levels of cyclin E2 RNA increase in mock infected cells. The fact that cyclin E2 protein levels do not show a similar increase at this time likely reflects the delay between transcriptional activation and protein translation.

## Discussion

These observations provide the first comprehensive identification of multiple cellular targets of a viral miRNA using RISC-IP analysis. Strikingly, the study demonstrates that miR-US25-1 mediates inhibition of gene expression through the novel mechanism of targeting 5′UTR sequences. Furthermore, we demonstrate that miR-US25-1 targets multiple cellular genes related to cell cycle control. To our knowledge this is the first example of a viral miRNA targeting 5′UTRs and is the first demonstration of an endogenous miRNA repressing protein expression through targeting sequences within the 5′UTR.

These results are in contrast to previous studies demonstrating miRNA targeting of 5′UTRs. Targeting of cellular 5′UTRs by miR-10a moderately increased gene expression while targeting of the HCV genome by miR-122 was shown to be required for viral replication [20], [21]. Clearly, targeting of 5′UTRs by miRNAs can mediate distinct positive or negative regulatory effects depending on the context. It will be interesting to determine how miRNAs mediate distinct regulatory effects and whether inhibition of protein expression through miRNA targeting of 5′UTRs is more common than previously thought, or whether this mechanism is specific to miR-US25-1 or viral miRNAs.

The functions of a number of cellular genes identified in this study have important implications for the biology of HCMV and viruses in general. Infection with HCMV has long been known to manipulate the cell cycle by altering the expression of cyclin dependent kinases (CDKs) and their associated cyclin subunits [27]. Cyclin E proteins are expressed early in G1 phase where they bind to and activate CDK2, resulting in progression into S phase. Previous studies have demonstrated that HCMV induces resting G0 cells to enter the cell cycle whereupon the virus blocks further progression at the G1/S boundary [34]. By blocking the cell cycle at the G1/S phase the virus creates a cellular environment conducive for DNA replication. HCMV induced expression of cyclin E1 is thought to play an important role in driving cells into the G1/S phase [35].

Here, we show the virus also induces expression of cyclin E2 early in infection, then moderates cyclin E2 protein levels through targeting by miR-US25-1. miR-US25-1 may therefore function as a rheostat regulator, modulating expression of cyclin E2 to generate the correct balance in protein induction. This may contribute to the viruses ability to block cell cycle progression at the G1/S phase, or to protect the infected cell against toxicity. Over-expression of cyclin E has been linked to sensitivity to apoptosis and unchecked induction of cyclin E2 may be detrimental to the virus [36], [37].

Alternatively, miR-US25-1 function may be unrelated to cell cycle control. Recent studies have suggested that herpesvirus miRNAs may be important during persistent or latent infection [9], [11]–[13]. HCMV is thought to reside within haematopoietic stem cell populations that give rise to latently infected monocyte and macrophage cells [38], [39]. By targeting genes involved in cell cycle progression and differentiation, the virus could manipulate the production of cells generated by latently infected progenitors to favor certain cell types such as monocytes and macrophages. Although deletion of US25-1 did not result in a phenotypic effect on the replication following infection of primary human fibroblast cells, regulation of the target genes identified may be important in other cell types, such as endothelial cells or macrophage cells, or during the latent or persistent phase of the virus life cycle.

Finally, the study of viral miRNAs may provide a powerful method for identifying novel cellular regulatory and antiviral pathways. This study suggests that viral miRNAs, like cellular miRNAs, may function through targeting multiple genes within related pathways. Many of target genes identified in this study are functionally related and contain the same 5′UTR sequence motif. It is likely that miR-US25-1 has evolved to target this 5′UTR motif, thereby subverting the regulatory pathway for the benefit of the virus. Investigation of viral miRNAs may therefore lead to discovery of additional novel cellular pathways.

## Materials and Methods

### RISC-IP analysis

RISC-IP analysis was carried as out previously described [22], [23]. In brief HEK293 cells stably transfected with c-myc tagged Ago2 were transfected with the pSIREN expression plasmid or a synthetic biotinylated siRNA using Fugene (Roche) or RNAimax (Invitrogen) according to manufacturers specifications. Three days post infection cells were lysed, samples taken for total RNA levels and miRNP complexes immunoprecipitated using anti-c-myc antibody beads (Sigma) or streptavidin beads. RNA was isolated using Trizol and analyzed for quality using an Agilent Bioanalyzer and transcript levels determined on the Illumina HumanRef-8 platform. Microarray data was analyzed using Gene sifter software. Enrichment of specific transcripts, through association with miRNP complexes was determined by dividing the immunoprecipitated levels of transcripts by the total levels, thereby taking into consideration any direct effects of miR-US25-1 on transcript levels. This approach initially identifies any transcript associated with any miRNP complex. To specifically identify those transcripts targeted by miR-US25-1, the enrichment profile was compared to cells transfected with a negative control vector, resulting in exclusion of transcripts enriched through association with cellular miRNAs. Transcripts were then ranked according to the level of enrichment with the highest enriched transcripts considered potential targets of miR-US25-1. For example, in cells transfected with the negative control plasmid, cyclin E2 levels were 1958 in the total sample and 2219 in the IP sample, giving an enrichment value of 1.1. In cells transfected with miR-US25-1 expression plasmid, cyclin E2 levels were 2718 in the total sample compared to 16744 in the IP sample, giving an enrichment value of 6.1. By dividing the enrichment value from cells transfected with US25-1 compared to the control cells (6.1/1.1) the overall enrichment ratio is calculated as 5.4.

Argonaute specific antibody was generated by immunization of rabbits with a peptide corresponding to the N terminal region of Argonaute 2 (5-MYSGAGPALAPPAPPPPIQGYAFKPPPRPD3′). For virus infections, primary human fibroblast cells (Clontech) were infected at high multiplicity (MOI of 10) with the laboratory lab strain AD169. RISC-IP analysis was conducted as above, except antibody to endogenous Ago2 was used to immunoprecipitated miRNP complexes and transcript levels determined using direct RT-PCR primer probe sets (ABI) for CCNE2, TRIM28, and GAPDH.

### Sequence analysis

Transcript sequences were down loaded from NCBI using RefSeq ID's. Transcript data sets were searched for seed sequence matches using a Java based script program. Statistical overrepresentation of seed matches within the top 50 transcripts from Table S3 was determined by Fisher exact test. Predicted binding between miR-US25-1 and target sites within the 10 most highly enriched transcripts were determined using the online RNA folding algorithm, mfold (http://mfold.bioinfo.rpi.edu/cgi-bin/rna-form1.cgi).

### Luciferase assay

5′UTRs and approximately 500 bases upstream sequence of target genes were PCR amplified from human fibroblast DNA and cloned upstream of the pGL4 luc2 (Promega) firefly luciferase construct. 5′UTR luciferase constructs were cotransfected with a renillin control construct and miRNA mimics using Lipofectamine 2000 (Invitrogen) into HEK293 cells according to manufacturers instructions. Cells were harvested 18 hours post transfection and luciferase levels measured using Promega's dual reporter kit. Protein levels were determined using an anti-firefly luciferase antibody (Sigma).

### Western blot analysis

Human primary fibroblast cells were grown in either 10% serum or 0.01% serum for 18 hours before infection at high multiplicity (MOI of 10) with either wild type AD169 or miR-US25-1 knock out virus. Serum rescued cells were recovered with 10% serum 48 hours post infection. Seventy-two hours post infection, cells were harvested using RIPA buffer and total protein levels determined by BCA analysis. Thirty micrograms total protein was loaded and proteins detected using primary antibodies to cyclin E2 (Abcam), TRIM28 (Cellsignal), and GAPDH (Abcam) and secondary HRP-conjugated antibodies (Jackson labs) with ECL reagent (GE bioscience).

### miRNA expression cassettes

The predicted pre-miRNA region of miR-US25-1 plus approximately 100 additional bases were PCR amplified (primers shown in supplementary Table S4) and cloned into the pSIREN expression plasmid (Clontech). pSIREN NEG was supplied by Clontech. Synthetic miRNA mimics were purchased from Dharmacon.

### miR-US25-1 KO virus

miR-US25-1 pre-miRNA coding region was deleted from AD169 BAC clone using BAC technology as previously described [40]. Briefly a PCR amplified cassette containing FRT flanked Kanamycin was recombined into AD169 BAC genome replacing the miR-US25-1 coding region using primers listed in Table S4. The Kanamycin cassette was then removed by recombining the FRT sites through inducible FLIP recombinase. The resulting BAC was isolated and electroporated into human primary fibroblast cells to produce infectious virus.

### RT-PCR analysis

Total RNA was harvested using Trizol and concentrations determined on a nano-drop spectrophotometer. 100 ng of total RNA was then reverse transcribed using either random hexemers or specific RT primers for miRNA RT-PCR. Specific primer probe sets were then used for real time amplification using TAQMAN probes. All primers and probes shown in Table S4. Gene specific primer probe sets were from ABI.

## Supporting Information

Figure S1 Predicted miR-US25-1 target sites in enriched transcripts. Schematic representation of enriched transcripts showing position and predicted base pairing of miR-US25-1 to binding sites. ORFs are shown in red with UTRs shown in green. Seed regions are highlighted in red. miRNA binding was predicted using mfold.
(1.33 MB EPS)

Figure S2 (a) To confirm the effectiveness of the generated antibody, HEK293 cells were transiently transfected with Ago1, 2 or 3 expression plasmids, or mock transfected (M), and protein extracts analyzed by western blot analysis. The generated antibody reacted strongly with endogenous and transiently expressed Ago2, but not with Ago1 or 3. (b) The Ago2 antibody efficiently immunoprecipitates Ago2. 293 HEK cells stably expressing myc-tagged Ago2 were labeled with S35 methionine and immunoprecipitated with either 5, 10 or 15 ul of Ago2 antibody or 10 ul c-myc antibody (10 ul Ago2 antibody for denatured sample). Both denatured (D - lane 2) and non-denatured (N - lanes 3, 4 and 5) samples were Immunoprecipitated then separated on a 10% SDS gel and exposed to film overnight. Bands corresponding to Ago2 were detected with both Ago2 antibody and the c-myc antibody. No bands were detected using pre-bleed serum (Pre-bleed). (c) AD169 US25-1 KO virus grows with the same kinetics as wild type in primary human fibroblast cells infected at a multiplicity of 10 pfu per cell. (d) Levels of cyclin E2 were determined by western blot analysis 24, 48 and 72 hours following infection of primary human fibroblast cells with either wild type or US25-1 KO virus. Deletion of US25-1 resulted in higher levels of cyclin E2 only after 48 hours infection. GAPDH is shown as a loading control.
(3.00 MB EPS)

Table S1 Full data set for RISC-IP analysis of miR-US25-1 pull downs using myc-Ago2 approach. Signal levels for total RNA and IP RNA levels are shown for biological replicates A and B. IP/Total ratios were generated for NEG and miR-US25-1 transfected cells and enrichment determined by dividing miR-US25-1 ratio by NEG ratio. Enrichment from the biological replicates were averaged and genes sorted based on this value.
(5.70 MB ZIP)

Table S2 Full data set for RISC-IP analysis of miR-US25-1 pull downs using biotin approach. Data analyzed as for Table S1.
(5.51 MB ZIP)

Table S3 Data sets were combined by determining the average rank from Tables S1 and S2 based on average enrichment. Number and type of target within the 5′UTR is also shown for each gene. Transcripts containing miR-US25-1 target sites in top 50 are highlighted in yellow.
(2.53 MB ZIP)

Table S4 List of primers and probes used for cloning and RT-PCR analysis.
(0.05 MB DOC)
