# Supplementary material for: A Viral microRNA Down-Regulates Multiple Cell Cycle Genes through mRNA 5′UTRs
Source: PLoS Pathog. 2010 Jun 24;6(6):e1000967. doi: 10.1371/journal.ppat.1000967 (PMC2891821; doi:10.1371/journal.ppat.1000967)
Supplement: Table S4 — List of primers and probes used for cloning and RT-PCR analysis. (0.05 MB DOC) [file ppat.1000967.s006.doc]

| **Primer name** | **Sequence** |
| --- | --- |
| CCNE2 F | ATAGGTACCCTGGGGAGAGCAGAGCCG |
| CCNE2 R | GCGAAGCTTTCTCTTCTTTCAGGTGTA |
| H3F3B F | GCGGCTAGCAATGTATGTGCATGATA |
| H3F3B R | CGCCCATGGTTTCTTTCACCTAAGAA |
| miR-US25-1 F | GCGGATCCCGGCGGCCGGGGCTG |
| miR-US25-1 R | GCGAATTCAAAAAATTGATTTGCCTCGGTCTGAGAAC |
| US25-1 KO F | AAGCAACGCTACACCGTCACCCCGCTCCCAAGCGCCGCGGA*GTAAAACGACGGCCAGT* |
| US25-1 KO R | AGCGCTTCCCGCTTCGCCACCCCGCGCCCGCCCCAGTTT*CAGGAAACAGCTATGAC* |
| UL112-1 RT | GTCGTATCCAGTGCAGGGTCCGAGGTATTCGCACTGGATACGACAGCCTG |
| UL112-1 F | CGCGCAAGTGACGGTGAGAT |
| UL112-1 R | GTGCAGGGTCCGAGGT |
| UL112-1 Probe | ATACGACAGCCTGGAT |
| US25-1 RT | GTCGTATCCAGTGCAGGGTCCGAGGTATTCGCACTGGATACGACGGTCCG |
| US25-1 F | GCGCAACCGCTCAGTGGC |
| US25-1 R | GTGCAGGGTCCGAGGT |
| US25-2 Probe | TGGATACGACGGTCCG |

Supplemental Table 4. List of primers and probes used for cloning and RT-PCR analysis.
